# The mitochondrial genome of *Apion squamigerum* (Coleoptera, Curculionoidea, Brentidae) and the phylogenetic implications

**DOI:** 10.7717/peerj.8386

**Published:** 2020-01-13

**Authors:** Nan Song, Xinxin Li, Xinming Yin, Xinghao Li, Shengjun Yin, Mingsheng Yang

**Affiliations:** 1 College of Plant Protection, Henan Agricultural University, Zhengzhou, China; 2 Department of Chinese Medicine, The Second Hospital of Tianjin Medical University, Tianjin, China; 3 College of Life Science and Agronomy, Zhoukou Normal University, Zhoukou, China

**Keywords:** Mitogenome, Brentid beetle, Large intergenic spacer, Phylogeny

## Abstract

In this article, we present the nearly complete mitochondrial genome (mitogenome) of the weevil beetle *Apion squamigerum* (Curculionoidea, Brentidae), assembled using data from Illumina next generation sequencing (NGS). This mitogenome was found to be very large, with the total length of 18,562 bp. Two *trnM* genes were identified. A large non-coding intergenic spacer spanning 1,949 bp occurred between *trnI* and trn*M2*. Combined with 111 existing weevil mitogenomes, we conducted phylogenetic reconstructions based on various datasets under maximum likelihood and Bayesian inference. Firstly, phylogenetic analyses robustly supported a sister group of *A. squamigerum* and *Rhopalapion longirostre*, namely, that two species of Apioninae (Brentidae) formed a clade. Within the entire Curculionoidea, the Nemonychidae diverged firstly, following the families Anthribidae and Attelabidae. In addition, a large clade comprising the sister families Brentidae and Curculionidae was strongly supported in all trees.

## Introduction

Weevil beetles (Curculionoidea), with approximately 62,000 species in 5,800 described genera, are one of the most diverse groups in the order Coleoptera (Oberprieler, Marvaldi & Anderson, 2007). This group of beetles are characterized by a head extended into a proboscis. They are all plant-feeders. Some species are considered as quarantine pests due to their potential harms to the native plants. The other weevil species are perceived as beneficial, or of no importance in plant production. The diversification of weevil beetles has been often ascribed to co-radiation with the angiosperms (Farrell, 1998; McKenna et al., 2009). However, the explanatory account of the diversification of Curculionoidea based on the studies of combining higher-level phylogenies of weevils with host plant information may be limited by inadequate taxon sampling (Franz & Engel, 2010). The ancient origin of weevils (McKenna et al., 2009) and the extremely high species richness have made the phylogenetic reconstruction of this group challenging. In particular, the basal relationships within Curculionoidea remain elusive.

Weevils (Curculionoidea) are conventionally classified into two groups: Orthoceri, and Gonatoceri (Kuschel, 2003; Skuhrovec, 2007; Haran, Timmermans & Vogler, 2013). The Orthoceri contained the relatively primitive weevil families (Kuschel, 2003), such as Nemonychidae, Belidae, Anthribidae, Attelabidae and Brentidae. This set of families have straight antennae, which separate them from the “true weevils” Curculionidae that are characterized by geniculate antennae. The Gonatoceri included the advanced weevils, of which the Curculionidae constitute the largest family of Curculionoidea, containing 51,000 described species distributed over 4,600 genera (Oberprieler, Marvaldi & Anderson, 2007; Bouchard et al., 2011; Oberprieler, 2014). In addition to the geniculate antennae, most of true weevils also have the male genitalia with a fused pedon and tectum, which distinguished from the orthocerous weevils. However, the characteristic male genitalia associated with orthocerous weevils were also recognized in Rhynchophorinae (Curculionidae) (Morimoto, 1962). Further investigations on the genitalia morphology led to the establishment of some family-level groups, for example, the Brentidae (including subfamilies of Brentinae, Apioninae, Ithycerinae, Microcerinae, Nanophyinae) and Brachyceridae (with subfamilies Brachycerinae, Eririhininae, Cryptolarynginae, Raymondionyminae, Ocladiinae) (Bouchard et al., 2011). The interrelationships of the major groups of Curculionoidea are still controversial.

In the system of Bouchard et al. (2011), the superfamily Curculionoidea is divided into nine extant families: Nemonychidae, Anthribidae, Attelabidae, Belidae, Brentidae, Caridae, Dryophthoridae, Brachyceridae, and Curculionidae. The more recent study by Oberprieler (2014) recognized seven major lineages of weevils (Nemonychidae, Anthribidae, Attelabidae, Belidae, Brentidae, Caridae, and Curculionidae). In the latter, palm weevils (Dryophthorinae) and brachycerid weevils (Brachycerinae) are considered as subfamilies in Curculionidae. In addition, an eight-family system has been suggested by a phylogenomic study of Shin et al. (2017). The Cimberidinae formerly as a subfamily of Nemonychidae was elevated to the family rank (Shin et al., 2017). The study of Haran, Timmermans & Vogler (2013) with mitogenomic data also recovered Cimberidinae as a sister group to all other weevils. Within Curculionoidea, a stable sister group relationship between Brentidae and Curculionidae has been indicated (Marvaldi & Morrone, 2000; Oberprieler, 2000; Marvaldi et al., 2002; Shin et al., 2017).

Mitochondrial genome (mitogenome) sequences have been widely used to the phylogenetic reconstructions of insects (Cameron, 2014a). As a class of molecular marker, mitogenome has proved to be a useful source of information on the relationships at the level of families and superfamilies in Coleoptera (Timmermans & Vogler, 2012; Gillett et al., 2014; Crampton-Platt et al., 2015; Yuan et al., 2016). However, the number of mitogenome sequences available for Brentidae is vary sparse, and only four partial mitogenomes (10,629 bp∼12,664 bp) have been reported in GenBank to date (Haran, Timmermans & Vogler, 2013; Gillett et al., 2014). Additional mitogenomic data are needed to elucidate the phylogeny of Brentidae and to resolve the higher-level relationships in Curculionoidea.

In this study, we determined a nearly complete mitogenome of Brentidae, *Apion squamigerum*, with an NGS based approach. The detailed description of genome organization is presented. Combined with published beetle mitogenome sequences, we reconstructed the phylogenetic relationships in Curculionoidea, based on various datasets under maximum likelihood and Bayesian inference.

## Materials and Methods

### Specimen and DNA extraction

Adult specimen of *A. squamigerum* was collected 2015 in Jigong Mountain, Henan Province, China (the geospatial coordinates: 31.46°N, 114.01°E). No specific permits were required for the insect sampled for this study. The sample was directly killed and preserved in absolute ethanol. It was stored at −20 °C until DNA extraction. Specimen identification was conducted by checking adult morphological characters (Kuschel, 2003; Fägerström, 2006), and blasting mitochondrial *cox1* gene fragment in public databases BOLD (Barcode Of Life Database: http://www.boldsystems.org- Identification section) and NCBI.

Total genomic DNA was extracted from a single specimen, wtih the TIANamp Micro DNA kit (TIANGEN BIOTECH CO., LTD) according to the manufacturer’s protocol. DNA concentration was determined using Nucleic acid-protein analyzer (QUAWELL TECHNOLOGY INC., Sunnyvale, CA, United States). After DNA extraction, the specimen parts have been deposited in Entomological Museum of Henan Agricultural University (voucher number: EMHAU-2015-Zz091005).

### Library preparation and sequencing

Genomic DNA were sent to Shanghai OE Biotech CO., LTD for library preparation and high-throughput sequencing. Library was constructed by using the Illumina TruSeqTM DNA Sample Prep Kit (Illumina, San Diego, CA, USA), with the insert size of 350 bp. The genome sequencing was conducted on an Illumina HiSeq2500 platform, with a strategy of 150 paired-end sequencing.

NGS QC toolkit (Patel & Jain, 2012) was used to filter raw data for quality control. The high-quality reads (avg. Q20 >90%, and avg. Q30 >80%) were used to assemble the mitochondrial contig, with the software Mitobim v1.9 (Hahn, Bachmann & Chevreux, 2013). In prior, the mitochondrial *cox1* gene fragment (5′-end, about 500 bp sequence) was sequenced as a starting reference. Both the PCR and Sanger sequencing reactions were conducted using the primers of Song et al. (2016). We employed Geneious R11 (Kearse et al., 2012) to perform read mapping to check the quality of the mitogenome sequences assembled.

We also used ARC (Hunter et al., 2015) and Geneious R11 (Kearse et al., 2012) to perform the reference-guided assemblies. The reference sequence was the mitogenome from the closely related brentid beetle species (*Rhopalapion longirostre*: Haran, Timmermans & Vogler, 2013). For the assembler of ARC, the configure input file was made with default settings. The parameter settings for assembly with Geneious R11 were identical to those in Yang et al. (2018).

### Mitogenome assembly and annotation

**Figure 1:**
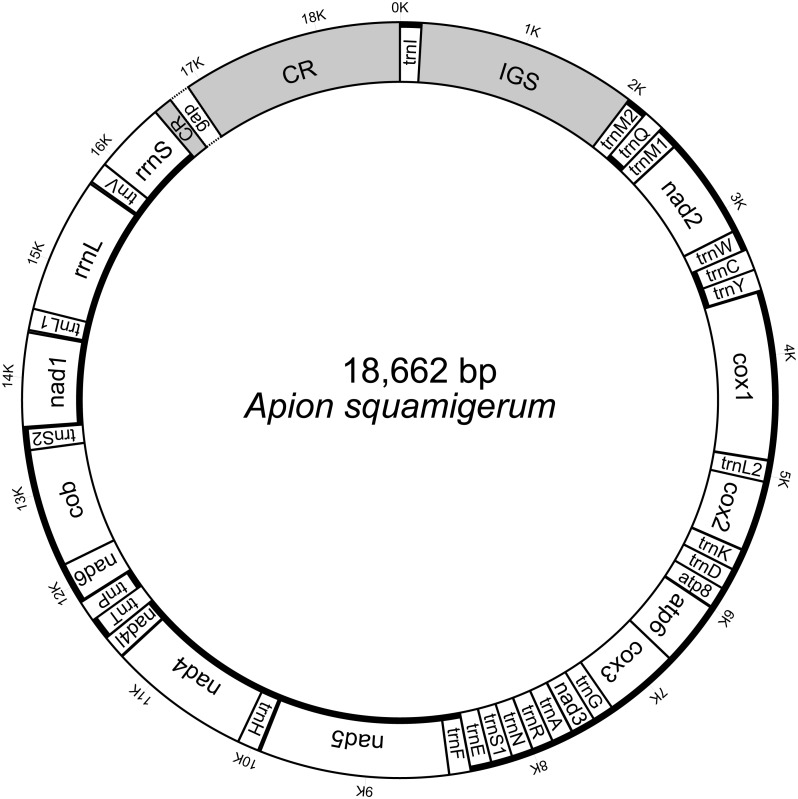
The structure of the mitochondrial genome of *Apion squamigerum*.

The initial mitogenome annotation was conducted in MITOS web (Bernt et al., 2013). The start codon, stop codon and length of each protein-coding gene were manually checked and adjusted by alignment to the published brentid beetle mitogenome sequences (see details in Table S1). The secondary structures of 22 tRNA genes were predicted by MITOS. The gene boundaries of two rRNA genes were refined by aligning against the published sequences. The corresponding secondary structures were predicted with reference to *Gonocephalum outreyi* (Coleoptera, Tenebrionidae) (Song et al., 2018). The genome structure images were generated using Mtviz (http://pacosy.informatik.uni-leipzig.de/mtviz) (Fig. 1) and OGDRAW (Greiner, Lehwark & Bock, 2019) (Fig. S1). The newly determined mitogenome sequence (File S1) of *A. squamigerum* has been submitted to GenBank under the accession number MN459662.

### Sequence alignment

Each protein-coding gene was aligned individually using TranslatorX (Abascal, Zardoya & Telford, 2010), with the following parameters: Genetic code = “invertebrate mitochondrial”, Protein alignment = “MAFFT”, and the stop codons excluded. The 22 mitochondrial tRNA genes and two rRNA genes were separately aligned using the program MAFFT under iterative refinement method incorporating the most accurate local (E-INS-i) pairwise alignment information (Katoh & Standley, 2013). The alignments were checked in MEGA 7 (Kumar, Stecher & Tamura, 2016). Poorly aligned sections were eliminated by Gblocks (Talavera & Castresana, 2007). Finally, the individual alignments were concatenated to make the datasets of PCG (nucleotide alignment including 13 protein-coding genes), PCG_AA (amino acid alignment including 13 protein-coding genes) and PCGRNA (nucleotide alignment including 13 protein-coding genes, two rRNA genes and 22 tRNA genes), with the Perl script FASconCAT_v1.0 (Kuck & Meusemann, 2010). The relative synonymous codon usages of protein-coding genes of *A. squamigerum* were examined by MEGA 7 (Kumar, Stecher & Tamura, 2016). The sequence alignments supporting the phylogenetic results of this article are presented in the File S2.

### Phylogenetic inference

In the phylogenetic analyses, our taxon sample included 112 weevil species representing five families of Curculionoidea, namely Nemonychidae, Anthribidae, Attelabidae, Brentidae, and Curculionidae. Following the classification of Oberprieler (2014), the families Belidae and Caridae are missing due to unavailability of mitogenomic data. In addition, five mitogenome sequences from Chrysomeloidea were selected as outgroups (Kastally & Mardulyn, 2017).

Phylogenetic trees were calculated using IQ-TREE (Nguyen et al., 2015) for maximum likelihood (ML) analyses and MrBayes 3.2.6 (Ronquist et al., 2012) for Bayesian inferences. PartitionFinder 2 (Lanfear et al., 2016) was used to select the optimal sets of partitions. Data blocks were predefined by genes for each dataset. The PartitionFinder analyses were run using a greedy search scheme (Lanfear et al., 2012), with all models considered under the Akaike information criteria.

ML searches were performed using IQ-TREE implemented in the Cipres Science Gateway (Miller, Pfeiffer & Schwartz, 2010). Data partition schemes pre-determined by PartitionFinder were used as inputs (Table S2), and substitution models were estimated *de novo* across all available models by ModelFinder (Kalyaanamoorthy et al., 2017) implemented in IQTREE. Allowing partitions to have different speeds (-spp) was selected for each ML analysis. Nodal support values (BP) were evaluated through an ultrafast bootstrap approach (Minh, Nguyen & Von Haeseler, 2013), with 10,000 replicates.

Bayesian analyses using MrBayes 3.2.6 (Ronquist et al., 2012) were conducted in the CIPRES Science Gateway (Miller, Pfeiffer & Schwartz, 2010). We applied the MrBayes blocks for partition definitions generated from PartitionFinder, which include the partition schemes (same as ML analyses) and the best-fitting models (nst = 6 rates = invgamma and/or nst = 6 rates = gamma for DNA, or Mtrev for protein). All model parameters were set as unlinked across partitions. Each analysis involved two independent runs, and started from random topology. Each run implemented four Markov chain Monte Carlo chains in parallel for at least 5,000,000 generations, and sampled every 1,000 generations. The program Tracer 1.7 (Rambaut et al., 2018) was used to analyze the trace files from two Bayesian MCMC runs. Sufficient sampling was believed to occur when the ESS value was above 100. In addition, we checked the raw trace plot to see if the chain was long enough for convergence. The first 25% of sampled trees were discarded as burn-in, and the remaining trees were used to calculate a 50% majority-rule consensus tree. Branch support was assessed by clade posterior probabilities (PP).

## Results

### Next-generation sequencing output and mitochondrial genome organization

As for the new mitogenome sequence of *A. squamigerum*, a total of 4,412,164 bases (about 29,416 mitochondrial reads) were mapped to the original 18,562 bp mitochondrial contig. The mean base coverage of the mitochondrial contig was 238-fold. The distribution of reads along the mitogenome was basically uniform. However, there were sharp decline at both ends. This may lead to the failure of assembling the complete control region.

With the reference mitogenome of *R. longirostre*, Geneious and ARC yielded the shorter mitogenome contig lengths, 11,355 bp and 12,507 bp, respectively. The reference sequence of *R. longirostre* is a partial mitogenome, with only 11,152 bp. The incomplete reference mitogenome led to a short mapping assembly. Both Mitobim and Geneious successfully assembled in a single mitochondrial contig. Whereas ARC produced three mitochondrial contigs, with lengths of 7,594 bp, 3,681 bp and 1,232 bp, respectively. The 7,594 bp contig had five nucleotides overlapping with 3,681 bp contig. There were 12 bp missing gap between 3,681 bp contig and 1,232 bp contig. Alignments showed that the sequences assembled from Geneious and ARC were identical to that obtained from Mitobim.

The nearly complete mitogenome of *A. squamigerum* consists of the 13 protein-coding genes, 23 tRNA genes, two rRNA genes and a putative control region (Fig. 1). There are 24 genes encoded on the heavy strand, while the remaining 14 genes encoded on the light strand. On the heavy strand, the second *trnM* gene (*trnM2*) is located adjacent to the 5′end of the *trnQ*. The typical *trnM1* occurs between *trnQ* and *nad2*. Four mismatched bases are detected between *trnM2* and *trnM1*. The nucleotide composition of the whole mitogenome of *A. squamigerum* is 39.1% A, 37.7% T, 13.2% C, 10.0% G. This result shows a strong bias towards A+T content (76.8%). AT skew is calculated by AT-skew = (A − T)/(A + T) and GC-skew is defined by GC-skew = (G − C)/(G + C). As a result, the AT-skew is 0.018, while the GC-skew is −0.138.

### Protein-coding genes and codon usage

The protein-coding genes excluding stop codons have a total length of 11,061 bp, which encodes 3,687 amino acid residues. All protein-coding genes started with the typical ATN codons, such as ATT for *nad2, cox1, cox2, nad3, nad5*, *nad6*, ATG for *atp6, cox3, nad4, nad4l*, *cob*, ATC for *atp8*, and ATA for *nad1*. Except for *nad5* and *nad4*, the remaining 11 protein-coding genes were inferred to terminate with the complete stop codon (TAA or TAG). Both *nad5* and *nad4* used TA as the stop codon.

**Figure 2:**
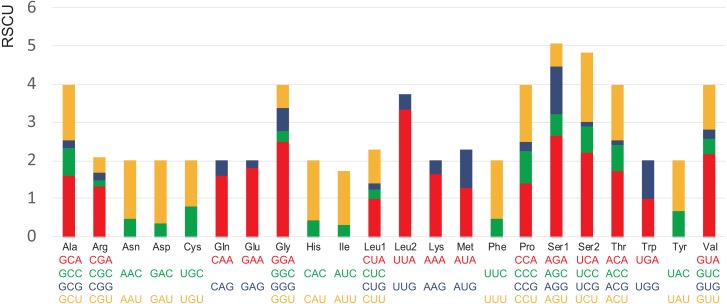
Relative synonymous codon usage (RSCU) in the *Apion squamigerum* mitochondrial genome.

The codon usage pattern of *A. squamigerum* mitogenome is shown in Table S3. In *A. squamigerum* mtDNA protein-coding genes, Ile (I), Asn (N), Leu2 (L2), Phe (F), Lys (K) and Met (M) are among the most frequently found amino acids with the frequency of AUU (7.5%) and AUC (1.6%) for Ile, AAU (6.7%) and AAC (2.1%) for Asn, UUA (7.7%) and UUG (0.9%) for Leu2, UUU (6.1%) and UUC (1.8%) for Phe, AAA (6.3%) and AAG (1.3%) for Lys, and AUA (6.6%) and AUG (0.7%) for Met, respectively. The relative synonymous codon usage (RSCU) values also indicated that all the frequently used codons are A/T-rich (Fig. 2). The A+T content of protein-coding genes was 73.0%, and the third codon positions had the highest A+T content (84.1%).

### Transfer RNAs and Ribosomal RNAs

**Figure 3:**
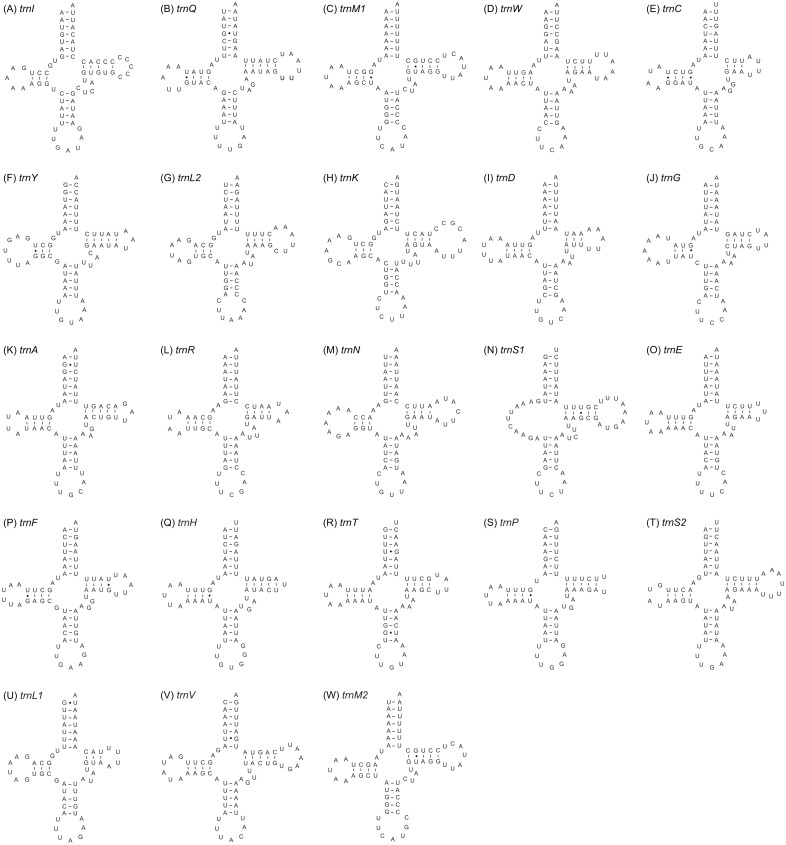
The inferred secondary structures of the 23 tRNA genes from *Apion squamigerum* (A–W).

Twenty-three tRNA genes were identified in the mitogenome of *A. squamigerum* and ranged in length from 58 bp to 70 bp. As mentioned above, the *trnM* with 69 bp in length occurs twice in the mitogenome. The inferred secondary structures for tRNA genes are provided in Fig. 3. All tRNA genes can be folded into the cloverleaf secondary structure, with the exception of *trnS1*. The *trnS1* lack a dihydrouridine (DHU) arm, which was replaced by a simple loop.

The large ribosomal gene (*rrnL*) is 1,270 bp in length, which was found between *trnL* (CUN) and *trn V*. The small ribosomal gene (*rrnS*) is 758 bp, and positioned between *trnV* and the control region. The secondary structures of both *rrnL* and *rrnS* are shown in Figs. S2 and S3. The secondary structure of *rrnL* contained five domains (labeled I, II, IV, V and VI) and 50 helices. Domain III was absent. The *rrnS* gene was composed of three domains (labeled I, II, III) and 27 helices.

**Figure 4:**
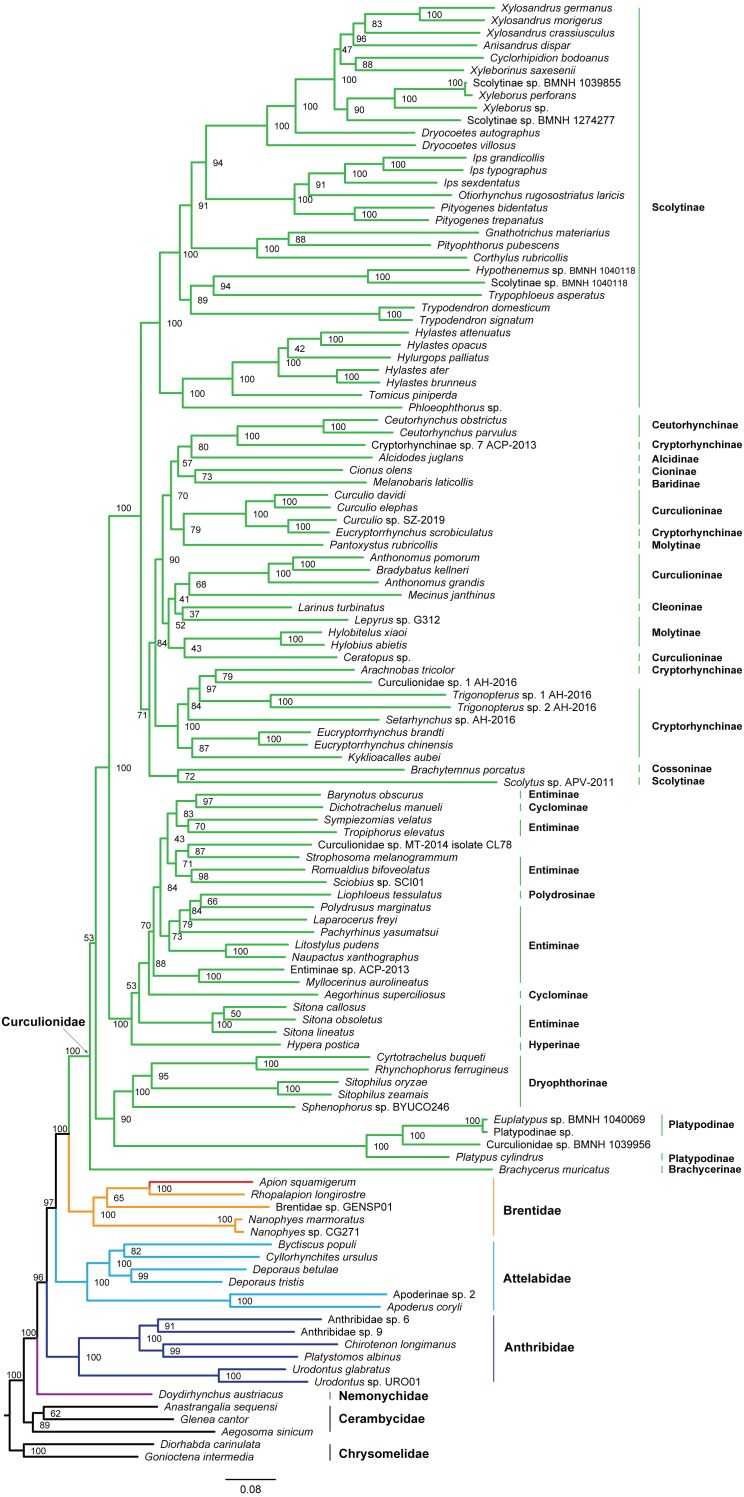
Maximum likelihood tree inferred from the dataset of PCGRNA using IQ-TREE.

### Control region

In the assumed position corresponding to the control region (i.e., between *rrnS* and *trnI*), two prominent non-coding regions were assembled at both ends of the original mitochondrial contig. There were no overlapping regions found between two sequences. The A+T contents of two fragments corresponding to the control region are 86.3% (153 bp in length) and 82.8% (1,768 bp in length), respectively. That is obviously higher than the A+T content of the entire mitogenome (76.8%). Although there is no obvious tandem repeat unites identified, the [TA(A)]n-like sequence occurs many times in the partial control region assembled.

**Figure 5:**
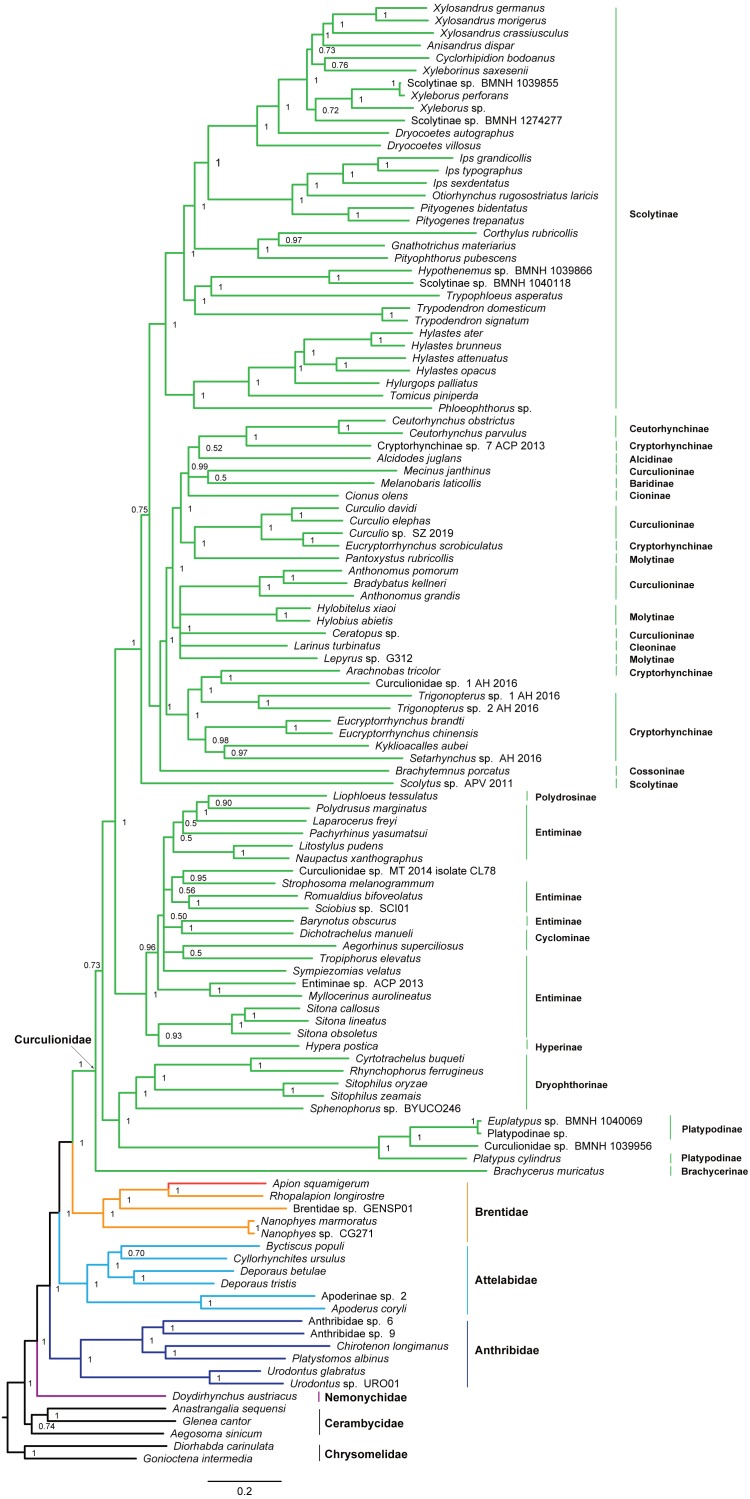
Bayesian tree inferred from the dataset of PCGRNA using MrBayes.

### Phylogenetic analyses

The superfamily Curculionoidea and five of its family-level lineages (Nemonychidae, Anthribidae, Attelabidae, Brentidae and Curculionidae) were strongly supported (BP ≥ 99, PP = 1.0) across all of our analyses (Figs. 4 and 5 and Figs. S4–S7). The Nemonychidae, represented by a single species of *Doydirhynchus austriacus*, was placed as a sister group to all other weevils. In the nucleotide analyses (Figs. 4 and 5 and Figs. S4–S5), the Anthribidae formed the second splitting group, and followed by the Attelabidae. By contrast, the Attelabidae branched before Anthribidae in the amino acid trees (Figs. S6–S7). The Brentidae was consistently placed as a sister group to Curculionidae in all analyses (BP ≥ 98, PP = 1.0).

Within Brentidae, the newly sequenced *A. squamigerum* was robustly supported as a sister group to another species of Apioninae (*Rhopalapion longirostre*). Among the rest of brentid beetles, two representatives of Nanophyinae formed a sister group. The classification of the exemplar of Brentidae sp. GENSP01 was ambiguous. The current taxon sampling was limited to draw conclusions regarding the subfamily relationships in Brentidae.

Within the family Curculionidae, the Brachycerinae, Platypodinae and Dryophthorinae were recovered as the basal lineages. The relationships among the remaining curculionid subfamilies were different across analyses. Most of the curculionid subfamilies represented by multiple exemplars (Entiminae, Scolytinae, Cryptorhynchinae, Curculioninae and Cyclominae) were retrieved as non-monophyletic.

## Discussion

### General features of *A. squamigerum* mitogenome

For the mitogenome of *A. squamigerum*, the positions and orientations of protein-coding genes, ribosomal RNA genes, tRNA genes, and the putative control region are consistent with the hypothesized ancestral insect (Cameron, 2014a), except for the presence of the second *trnM* gene and a ‘supernumerary’ large non-coding region. The analyses of AT-skew and GC -skew values show that the mitogenome of *A. squamigerum* has roughly equal A and T composition. There is a strand asymmetry in the distribution of G and C. The heavy strand is skewed toward C content. These data are congruent with the usual strand bias of metazoan mtDNA (positive AT-skew and negative GC-skew for the heavy strand, (Dermauw et al., 2009).

Two protein-coding genes of *nad5* and *nad4* end at incomplete (i.e., TA) codons., which can be completed via posttranscriptional polyadenylation (Ojala, Montoya & Attardi, 1981). The *trnS1* gene has an unpaired stretch of 11 nucleotides leading to the absence of the DHU arm (Fig. 3N). For the *rrnL* gene, Domain III is missing in the inferred secondary structure. These patterns are also found in most of other published insect mitogenomes (Cannone et al., 2002; Cameron, 2014a; Cameron, 2014b).

The only gap in the mitogenome of *A. squamigerum* occurs in the control region. We tried to close this gap region using PCR amplification and Sanger sequencing. Unfortunately, amplification and sequencing failed due to the degeneracy of primers and the poor DNA quality. Cameron (2014b) suggested that it is often impossible to determine the complete sequence of the insect control region by PCR amplification and Sanger sequencing. Because this region has some characteristic structural properties, such as the significantly high A+T content, the stretch of poly A or poly T, and tandem repeats (Cameron, 2014b). These characteristics make it difficult to design useful primers for sequencing. Even with NGS methods, it is still a challenge to reconstruct the control region under both mapping and assembly softwares (Crampton-Platt et al., 2015).

### The ‘supernumerary’ large non-coding region

The animal mitogenome is typical very small in size, with length of 15–17 kb and few non-coding nucleotides (Wolstenholme, 1992). Variation in mitochondrial size is generally a consequence of variation in the length of the non-coding region (Thao, Baumann & Baumann, 2004). In this paper, the nearly complete mitogenome of *A. squamigerum* has a genome length of 18,562 kb, which is larger than average insect mitogenomes. Besides the control region described above, the presence of a 1,949 bp ‘supernumerary’ non-coding region between *trnI* and *trnM2* (namely the IGS in Fig. 1) contributes to the larger size of *A. squamigerum* mitogenome.

Comparison across weevil mitogenomes published, several species in the family Curculionidae also have a genome length than 18 kb. Moreover, a ‘supernumerary’ large non-coding region occurred in the mitogenomes of *Eucryptorrhynchus scrobiculatus* (Cryptorhynchinae), *Curculio* sp. SZ-2019 (Curculioninae), *Sitophilus zeamais* (Dryophthorinae), *Pantoxystus rubricollis* (Molytinae), *Ips sexdentatus* and *Pityogenes bidentatus* (Scolytinae) (Fig. S1). The large non-coding region (except the control region) was also found in other beetle lineages, for example, the families Cerambycidae (Wang et al., 2019) and Coccinellidae (Song et al., in press). The possible evolutionary mechanisms behind the ‘supernumerary’ large non-coding region include the slipped-strand mispairing and random loss model and the duplication/random loss model (Wang et al., 2019).

### Higher-level relationships of weevils

At the family level, the Nemonychidae was found to be a sister group to all other weevils. This arrangement was congruent with previous studies (Haran, Timmermans & Vogler, 2013; Timmermans et al., 2015; Shin et al., 2017). The Anthribidae and Attelabidae were placed in an intermediate position between the Nemonychidae and a large assemblage comprising Brentidae and Curculionidae. However, the relative position of Anthribidae to Attelabidae varied depending on the dataset used. Both Brentidae and Curculionidae are angiosperm-associated groups (Shin et al., 2017), which indicates a close relationship between them. The analyses based on the mitogenome data consistently supported Brentidae as a sister group to Curculionidae. This result corroborates previous molecular studies (Marvaldi et al., 2009; Haran, Timmermans & Vogler, 2013; Gillett et al., 2014; Shin et al., 2017). The significantly statistical support for the major nodes in Curculionoidea demonstrates that mitogenome sequences may be useful in resolving deep divergences of weevil beetles.

The classification schemes for the family Curculionidae have been unstable (Haran, Timmermans & Vogler, 2013; Gillett et al., 2014; Gunter, Oberprieler & Cameron, 2016; Shin et al., 2017). Definitions of some subfamilies are considered as tentative (Shin et al., 2017). The Brachycerinae, represented by the sole species of *Brachycerus muricatus*, was retrieved as sister to the remaining Curculionidae in the trees inferred from the nucleotide dataset of PCGRNA (Figs. 4 and 5). This result supported the brachycerid clade as a distinct family-level lineage (namely Brachyceridae) (Gillett et al., 2014; Mckenna et al., 2015). In addition, PCGRNA dataset consistently recovered Platypodinae as a sister group to Dryophthorinae, with the significantly statistical support (BP = 90, PP = 1.0). This result are consistent with previous analyses based on morphological characters (Marvaldi, 1997) or molecular evidence (McKenna et al., 2009; Haran, Timmermans & Vogler, 2013; Shin et al., 2017; Mugu, Pistone & Jordal, 2018). The analyses of datasets of PCG and PCG_AA resolved the relationships differently, with Brachycerinae as the sister group to Platypodinae. Both Brachycerinae and Platypodinae were sister to Dryophthorinae (Figs. S4–S7). Considering the conflicting results between analyses and the non-monophyly of most subfamilies, additional data are needed to elucidate the positions of the brachycerid beetles and the subfamily relationships within Curculionidae.

## Conclusions

In the present study, we utilized NGS data to reconstruct the nearly complete mitogenome of the weevil beetle *A. squamigerum* (Brentidae). The current mitogenome sequences available for Brentidae are very limited. *A. squamigerum* mtDNA was only the fifth mitogenome annotated within 4,000 described species of Brentidae. This mitogenome is very large (18,562 bp), given the presence of the non-coding intergenic spacers spanning 1,949 bp. In addition, two *trnM* genes were identified. The presence of a large intergenic region and two *trnM* genes is interesting, further studies are needed to investigate the underlying mechanisms of the mitochondrial arrangements. The newly determined mitogenome is also expected to contribute to a better understanding of the phylogenetic relationships and evolutionary history of weevil beetles. The superfamily Curculionoidea and five families within it are consistently recovered. The major nodes received the significantly statistical support. These results suggest that the analysis of mitogenome sequences holds promise for the resolution of deep divergences of Curculionoidea.

##  Supplemental Information

10.7717/peerj.8386/supp-1 Table S1 Taxa included in this study

10.7717/peerj.8386/supp-2 Table S2 The best partitioning scheme selected by PartitionFinder and the corresponding models selected by both PartitionFinder and ModelFinder for the datasets of (A) PCGRNA, (B) PCG and(C) PCG_AA

10.7717/peerj.8386/supp-3 Table S3 The relative synonymous codon usages of protein-coding genes examined by MEGA 7

10.7717/peerj.8386/supp-4 Figure S1 Comparisons of mitogenome organization among six Curculionidae species

10.7717/peerj.8386/supp-5 Figure S2 Putative *rrnL* secondary structure in the *Apion squamigerum* mitogenome

10.7717/peerj.8386/supp-6 Figure S3 Putative *rrnS* secondary structure in the *Apion squamigerum* mitogenome

10.7717/peerj.8386/supp-7 Figure S4 Maximum likelihood tree inferred from the dataset of PCG using IQ-TREE

10.7717/peerj.8386/supp-8 Figure S5 Bayesian tree inferred from the dataset of PCG using MrBayes

10.7717/peerj.8386/supp-9 Figure S6 Maximum likelihood tree inferred from the dataset of PCG_AA using IQ-TREE

10.7717/peerj.8386/supp-10 Figure S7 Bayesian tree inferred from the dataset of PCG_AA using MrBayes

10.7717/peerj.8386/supp-11 File S1 The datasets (PCGRNA, PCG and PCG_AA) for phylogenetic reconstructions

10.7717/peerj.8386/supp-12 File S2 Format file of the *Apion squamigerum* mitogenome

## Additional Information and Declarations

### Data Availability

The following information was supplied regarding data availability: The newly determined mitogenome sequence of *Apion squamigerum* are available in GenBank: MN459662.
